# Dental Services Funding and Affordability in Serbia – Decade-Long Perspective

**DOI:** 10.3389/fpubh.2015.00145

**Published:** 2015-06-12

**Authors:** Tatjana Kanjevac

**Affiliations:** ^1^Faculty of Medical Sciences, Integrated Academic Studies of Dentistry, University of Kragujevac, Kragujevac, Serbia

**Keywords:** dentistry, health economics, costs, dental care, financing, affordability, reimbursement, Serbia

## Demand for Dental Care Services

Global awareness of growing demand for oral health care services became prominent over past several decades (1). Far reaching consequences of clinical dentistry for overall societal welfare were more obvious to the governing health authorities in mature market economies at first (2). Eastern European and Balkan health systems suffered from serious development setbacks during painful transitional health reforms taking place in the region since 1989 (3). Some of the core challenges across the region were temporary worsened insurance coverage in most countries of the region and huge contribution of out-of-pocket payments by ordinary citizens (4). As in so many areas of curative medicine, dental care was undergoing the same evolution. The early stages of this process were quite challenging for the regional health sector, and successful adaptation to the increasingly globalized health care market took many years to happen (5). Peculiarity of dental medicine is reflected in strong demand for frequent services, initiated by common acute disorders and adverse consequences of treatment procedures (6). Preventive and conservative dentistry in pediatric populations has significant long-term impact to the dental expenditure across the region (7). In some other areas, such as restorative dentistry, these issues are particularly prominent (8). In many transitional health systems, financial constraints worsened by global economic recession reflected heavily on dental care, imposing further narrowing of existing reimbursement practices as it happened in Bulgaria (9).

## The Case of Serbia

Serbia, as the largest successor state of former Yugoslavia, has its health system legacy in many ways different from Post-Semashko systems of other South East European countries (10). It is funded through one core state-owned health insurance fund (11) whose revenues mostly come from mandatory taxation of employers and employees (12). This transitional health system is currently undergoing significant reform from massive, hospital-based, supply-oriented one toward more responsive, lighter primary care-oriented system (13). Regardless of many successes, substantial challenges remain and these are reflected in evolving structure of national health care spending over past two decades (14). As in most of remaining European nations, work load to the entire health system and expenditures are dominated by accelerated population aging (15, 16) and prosperity diseases (17). So far, there are diverse difficulties related to inefficient funding mechanisms (18) and poor access and affordability of medical care (19) to the vast population of poor citizens and those residing in rural areas (20). Dental services remain seriously underfunded from public resources and this issue shapes the related to oral health of the nation (21). There are very few published local estimates on budget impact of clinical dentistry in the Balkans and related cost-of-illness studies. One of the few promising signs of growing awareness among policy makers is prioritizing oral health in some long-term national public health strategies (22).

## Public Spending for Dental Care and Medicines Over the Last Decade

**Figure 1:**
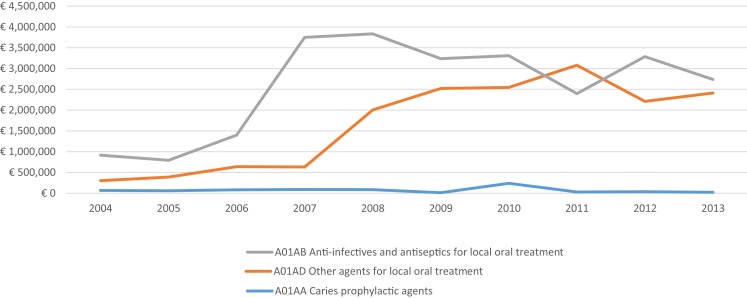
**Value-based turnover of publicly financed consumption of dental medicines presented across ground ATC medicines classes according to 2004–2013 growth in value-based turnover**.

Since 2004, National Agency for Medicines and Medical Devices of Serbia (ALIMS) issue commercially available periodic reports on precise structure of prescription dispensing and value of sales of all pharmaceuticals within the publicly funded health care facilities, pharmacies, and wholesalers. Recent research of trends over past decade has shown bold growth in local market size (23) dominated by biologicals (24) and oncology-related treatment options (25). These same sources offer us best attainable insight into the structure of Serbian market of dental medicines classified within broadly recognized Anatomical–Technological–Chemical classification system (ATC). Data presented in Figure 1 point out the falling prescription and dispensing of “Caries prophylactic agents” (A01AA code) whose value-based turnover decreased from € 66,704 in 2004 to € 21,397 in 2013 (actual peak value in the observed period was € 238,397 back in 2010). At the same time, sales of “Other agents for local oral treatment” (A01AD code) grew eightfold from € 302,608 in 2004 to € 2,413,302 in 2013 (actual peak value in the observed period was € 3,079,162 back in 2011). Slightly slower increase but the one dominating the market was the one of “Anti-infectives and antiseptics for local oral treatment” (A01AB code) rising from € 917,894 in 2004 to € 2,736,887 in 2013 (actual peak value in the observed period was € 3,833,995 back in 2008). Combined market size of all these three major groups of drugs used in various branches of dental medicine grew from € 1,287,207 in 2004 to € 5,171,585 in 2013. It is important to emphasize that entire national consumption of stomatological preparations actually contracted due to global economic recession and value of spending was exceeding € 6,000,000 back in 2010. The strong impact of economic crisis on Balkan pharmaceutical markets was noticed across the region with surprisingly better performance of transitional economies compared to OECD ones (26). Size of targeted public spending for oral health inclusive of capital investment, staff salaries, utilities, consumables, and other costs by far exceeds drug acquisition costs. According to the first officially available data, Republican Health Insurance Fund of Serbia (RFZO) has devoted € 51,131,383 in 2007 while over 21% less only 7 years after in 2013 (€ 40,351,340). Referring to the entire public health spending in the country funded by RFZO that this effectively meant percentage point decrease of governmental resources assigned for oral health programs from 2.82% in 2007 to 2.12% in 2013. These facts make de-investment into the clinical dentistry a rare exception compared to the many areas or clinical medicine regardless of crisis induced budget constraint (27). Such reimbursement policy imposed by local authorities effectively shifted financial burden of primary dentistry care to the ordinary citizens. Government legislature confirms that mandatory health insurance premiums were not inclusive of adult dentistry care unless in case of emergencies (28). Unlike among high-income EU economies, such policy in Serbia led to strong fall of demand for dental services motivated by simple lack of affordability rather than clinical need (29). Bureaucratic obstacles to the provision of dentist services contributed to the aforementioned phenomenon as well. Poor access to these medical services ultimately exposed well-known boomerang effect. Patients who were denied right to treatment in the early stages of their illness much later must be treated for severe form of neglected illness, which is much more expensive to treat. Outcomes of such delayed care are much less favorable and predictable and these interventions lose their cost-effectiveness when applied in clinically advanced cases (30).

## Core Future Challenges of Dental Services Provision and Financing

Dental medicine is one of the rare examples of flourishing of private-owned clinical facilities rising from historical legacy of state-owned health care in the region. Nevertheless, due to several core weaknesses, pace of contemporary Serbian oral health efforts seems to be insufficient to cover long-term population needs. Surprisingly, strong development of pharmaceutical market in other areas of clinical medicine occasionally reaches several fold annual growth. Due to public funding limitations, such setting creates significant pressures against financing of dental services. Accumulated public debt toward multinational industries of pharmaceuticals and medicinal devices will most likely continue to grow further (31). Some of the possible escape strategies should be rooted in evidence-based resource allocation policies. Regional efforts to establish feasible local health technology assessment agencies might be very rewarding in the long run. Throughout most of Western Balkans, cost-effectiveness estimates are not even mandatory condition for marketing approval of novel medicines. The core challenge lying ahead of more effective dental care provision in Serbia remains lack of insurance coverage and too high out-of-pocket payments by citizens. Bold growth of out-of-pocket health care spending is unfortunately evident in most globally leading emerging markets, which severely affects affordability of medical care to the poor (32). Demand for dentist’s services remains much higher than in most other clinical disciplines. Serbia’s dental healthcare market will probably achieve further growth in the upcoming years but mostly within its already conceived private sector. Decreasing public expenditure on oral health poses an unpleasant setback, which might be corrected after consolidation of economic growth (33). If national authorities commit themselves to prioritizing preventive dentistry (34), such move could yield significant gains (35). Unpredictable financial sustainability of existing health insurance systems in the Balkans will demand long-term efforts targeted to achieve accessible dental care for local communities in future.

## Conflict of Interest Statement

The author declares that the research was conducted in the absence of any commercial or financial relationships that could be construed as a potential conflict of interest.
